# Health Tract No. 330

**Published:** 1868-09

**Authors:** 


HEALTH TRACT No. 330.

### THE GUILLOTINE.

Was benevolently devised by a physician of that name for preventing all suffering on the part of those condemned to death ; and he himself, during the French Revolution, was executed by the instrument of his own devising. The body of a chicken bounces about for some time after the head has been cut or wrung off, showing a species of life there, but all feeling is in the brain, for if you cut the nerve leading to the brain from any part of the body, that part may be hacked to pieces without any discomfort being felt. When a certain brutal executioner held up the head of a lady and slapped the face, it crimsoned over as it would have done in life on the perpetration of such an indignity. If the head of an animal is cut off by a guillotine, there are signs of life for five minutes afterwards, "the nostril opens, the nerves twitch and the nose moves." When a person is dying sensation only ceases in a part when the blood cools ; this cooling process takes place slowly in the head because the most important vessels are in the interior which is protected and kept warm for some time by the thick bony covering of the skull ; hence we do not know but that by keeping the cut off head warm, its life might be protracted ; it may see and hear for hours ; there is then reason for believing that death by the guillotine, though apparently instantaneous, is one of the most dreadful, by reason of its protraction. The best way then is not to have the head cut off ; but go to the north pole and go to to sleep ; the most delicious feeling in nature is to be over-powered with sleep and to feel that you are at perfect liberty to do so ; on the other hand, it is really dreadful to be " so sleepy," under a dull sermon, and yet feel that you ought not to, and try not to, but oh what a bootless trial ! it is worse than having a dozen Augean Stables to clean ; the best antidote for a pulpit anodyne is to put a mite of red pepper in the corner of your eye ; it never fails.

